# Time Distances to Residential Food Amenities and Daily Walking Duration: A Cross-Sectional Study in Two Low Tier Chinese Cities

**DOI:** 10.3390/ijerph18020839

**Published:** 2021-01-19

**Authors:** Ziwen Sun, Iain Scott, Simon Bell, Xiaomeng Zhang, Lan Wang

**Affiliations:** 1 School of Design and Arts, Beijing Institute of Technology, Beijing 100081, China; 2 Edinburgh School of Architecture and Landscape Architecture, University of Edinburgh, Edinburgh EH3 9DF, UK; iain.scott@ed.ac.uk (I.S.); s.bell@ed.ac.uk (S.B.); 3 Chair of Landscape Architecture, Estonian University of Life Sciences, 51006 Tartu, Estonia; 4 Centre for Global Health, Usher Institute, University of Edinburgh, Edinburgh EH3 9DF, UK; xiaomeng.zhang@ed.ac.uk; 5 College of Architecture and Urban Planning, Tongji University, Shangai 200091, China; wanglan@tongji.edu.cn

**Keywords:** food environment diversity, food outlets, walking, street vending, age-related differences, Chinese neighborhood environment

## Abstract

Recent studies indicate the accepted concept of using land-use mix (LUM) to promote physical activity is ineffective and even counteractive in the Chinese context. Before considering LUM as a whole, different amenity types need to be respectively analyzed in relation to various functions and demands. This study aims to examine the specific associations between food-related amenities and perceived daily walking duration (WD) in small Chinese cities. Two interviewer-administered questionnaire surveys (*n* = 354) were conducted in Yuncheng and Suihua between 2017 and 2018. Logistic regression models were used to examine the associations of WD with seven different categories of food outlet at three levels of walking distance. The associations were further explored by food environment diversity and through two age groups. With the exception of café/tea house, the other six food outlets were positively associated with WD. After adjusting for socioeconomic variables, the associations of grocery store and supermarket weakened. Higher levels of food environment diversity were associated with a longer WD. Among the age groups, food outlets were more associated with older adults’ WD. This novel quantitative study suggests that increasing the number and heterogeneity of food-related amenities (including mobile street vendors) within a neighborhood can enhance physical activity in small Chinese cities.

## 1. Introduction

Globally, increasing physical activity (PA) has become an ever more significant topic for both research and practice due to its positive outcomes, such as reducing non-communicable diseases and promoting encounter opportunities to facilitate social relations [1,2]. World Health Organization (2018) reported that the residents who live in a walkable neighborhood are more physically active and contributes a healthier world, leading to the ontological turn from treatment to long-standing prevention in public health [3]. Numerous studies have proven that PA is associated with various factors, and some have refined several environmental attributes within a 3D framework (i.e., high “density”, land-use “diversity”, and pedestrian-oriented “design”) [4,5,6,7,8,9,10]. However, most of the existing studies have been conducted in developed countries, where the local contexts considerably differ in developing countries. For example, some recent studies have been conducted in Asia or South America. The results indicate that the associations of PA with land-use mix/diversity (LUM) and high residential density are ineffective or even counterproductive due to high speed of urban development, high-density sprawl, social-economic status, and informality [10,11,12,13,14,15]. This means that western theories and methods cannot be directly applied in China, and some validated factors need to be re-examined and disassembled.

Since the Chinese economic reforms of the late 1970s, in which bicycles and pedestrians once prevailed, have become dominated by cars, giving rise to a reduction in the amount of both walking and walkable space [16,17]. Many negative consequences have emerged, for example, between 2000 and 2014, the prevalence of obesity increased from 8.6% to 12.9% in a large population of 1.37 billion [18]. Under such urgent circumstances, designing walkable neighborhoods to increase public health has become a timely issue [8]. Over the last decade, some studies on walkability have been conducted in Chinese cities [10,11,13,17,19,20,21], but there are two issues in general. First, most of them have concentrated on large cities, with issues in small cities being largely under-represented where most Chinese people live in [14,22]. Due to factors in large cities such as better public transport infrastructure and higher levels of educational attainment [23,24], neighborhood environment impact on patterns of PA might differ in small cities. Secondly, these methods are mainly based on the application of western urban models, including Walkability Index [20], Walk Score [17,19], and Neighborhood Environment Walkability Scale (NEWS) [12,24], although some studies have adapted environmental factors according to the Chinese context.

More specifically, LUM is one of the key factors for increasing PA, and it can be measured the extent of heterogeneity in the distribution of amenities within a given area [25]. In Walkability Index and Walk Score, LUM is objectively measured via Geographic Information Systems, while in NEWS, it is about perceived walking proximity from participants’ home to the nearest amenities. Walkability Index only includes three land-use types (i.e., residential, retail, and office) and entropy value of LUM is often used for computation [10,26]. Walk Score considers 13 amenity types into five land use categories for measuring LUM: retail (e.g., grocery and book stores), educational (e.g., schools), food (e.g., restaurants), entertainment (e.g., theatres) and recreational (e.g., parks and gyms) [19]. These amenities are weighted according to their importance. However, objectively measured LUM might mismatch residents’ perception on the amenities of their neighborhoods [27]. NEWS involves 13 perceived amenity types but assuming that all destinations matter equally in the measurement of LUM [27,28]. 

Additionally, although some recent studies have refined amenities for measuring LUM based on different urban contexts, the common methods have inherently overlooked the nuanced associations of PA with different amenity types and land uses. For example, certain recreational areas in a neighborhood, such as urban park and blue space, have been positively related to PA and mental health, while it has been revealed that other recreational amenities (e.g., karaoke bar and cinema) might play a negative role in generating walking behaviors [12,29,30,31]. The official category of commercial land in China (Table 1) includes both cinema and restaurant, but they might have distinct associations with PA. Moreover, some amenities might have multiple functions and meanings in different contexts. For instance, in a food-related café of a small Chinese city, school children playing mobile phone games, and doing homework together at the weekend [12]. Therefore, we contend that before using LUM as a whole, different amenities need to be separately analyzed in relation to their different functions and demands in the local context.

Among different amenities, food providers are the most important demand for everyday life, and so can show us what matters in local contexts [33]. Many studies have examined associations of neighborhood food outlets with a series of outcomes, such as mortality [34], body mass index/obesity [35,36,37], unhealthy eating habits [38,39], age-related differences [37,40], socioeconomic status [35,41], neighborhood characteristics (e.g., school, worksite, or home) [41], and food intakes/purchase motivations [42,43,44]. Among different food-related outlets, a higher prevalence of obesity was found in areas that had small grocery stores or fast food providers, while lower in areas with supermarkets and fruit/vegetable providers [35,36,40,45]. However, few studies have examined the nuanced relations between proximity of different food outlets and PA in a neighborhood [28,44,46,47], and little empirical evidence has been gathered in small Chinese cities. 

The aim of this study, therefore, is to examine associations of perceived daily walking duration (WD) with neighborhood food outlets in low tier Chinese cities. Moreover, given the known differences in the role of Chinese urban neighborhood environment on walking behaviors by age [12], these associations are further explored through two age groups. Based on the above understanding, the research questions are as follows:

- How are the associations of the individual and the diversity of neighborhood food outlets with WD?
- Are the associations different between younger and older adults?

## 2. Materials and Methods

### 2.1. Study Area

Details of the definition of small Chinese cities are given elsewhere [12]. In short, according to the most recent Chinese census, the number of inhabitants in each of these two cities is around 5 million, and when Chinese cities are categorized via their urban population, 42.4% of the population of the entire country is shown to live in cities of 4 to 8 million [48,49]. The vast majority of the Chinese people are living in small and medium-sized cities. Based on the Chinese city ranking carried out by China Business Network Co. Ltd., the selected cities (Yuncheng and Suihua) belong to Tiers 4 and 5 out of five tiers [50]. This means that they are both small and are of the type that is commonplace and ordinary in China. Both cities are the prefecture-level municipality and have only one central district—in Yuncheng this is Yanhu district and in Suihua, Beilin district.

### 2.2. Study Design and Sampling

The questionnaire content with detailed items has been presented in another study [12], adapted from the NEWS-a short form [24]. We extracted three key subscales in accordance with the aim of this study, including perceived daily walking duration as the single outcome variable (WD) and seven types of food outlet (i.e., fruit/vegetable market, fruit/vegetable street vending, snack/breakfast street vending, convenience/small grocery store, supermarket, restaurant, and café/tea house). The third subscale included a range of socioeconomic characteristics: gender, age, educational attainment (junior college or lower and bachelor’s degree or higher), family income (3000 Yuan or less, 3001–5000 and 5001+) and occupation (employed, self-employed, and other). 

During the pilot study, field observation and open-ended interviews based on the questionnaire draft have been conducted in January 2017. Food outlets used in western studies [24,35] were adapted to the local contexts, such as street vendors and café/tea houses. A few studies in China noted that street vending could be an interesting factor to examine walkability [16,20,51]. The formal questionnaire surveys were conducted in the streets of the cities’ two central districts, in August 2017 (Yanhu) and June 2018 (Beilin), as people generally spend more time outdoors during warmer weather. Seven volunteers in Yuncheng were recruited from Yuncheng University and six volunteers in Suihua were recruited from the local urban planning department for the interviewer-administered approach. Volunteers attempted to ask every person on the city centre streets, although many busy pedestrians refused to participate in this survey. This research was approved by the University Ethics Committee (No. 06032017).

### 2.3. Data Collection 

**Figure 1:**
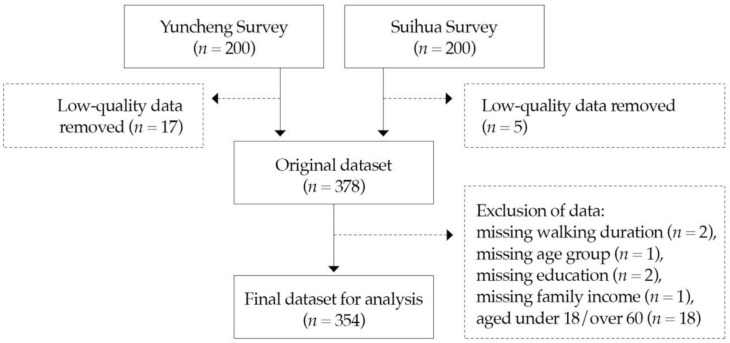
Flowchart of the data collection process.

We aimed to collect 200 questionnaires in each city, because the sample size of most previous studies on environment walkability was between 101 and 300 [52]. All data were carefully checked via a data-cleaning process [53]. The original datasets had 183 participants in Yuncheng and 195 in Suihua. After removing participants with missing walking duration (*n* = 2), missing age group (*n* = 1), missing education (*n* = 2) and missing family income (*n* = 1), and those aged under 18/over 60 (*n* = 18), the final datasets for analysis had 171 participants in Yuncheng and 183 in Suihua (Figure 1). Because of the small non-representative samples in the ordinary cities, we combined the two datasets (*n* = 354) and the city became an additional variable, which enabled regressions to be performed more feasibly.

### 2.4. Statistical Analysis

First, we categorized WD into two levels—greater than 60 min and equal to or less than 60 min—due to the recommended daily PA being at least 60 min and this duration as one of the cut points in many studies [25,28,54]. The walking distances to each amenity were categorized as 1–5 min, 6–10 min, over 10 min and missing [24]. “Missing” indicates “I do not know where the amenity is”. Stata 15 software was used to analyze the pooled dataset. We performed univariate and multivariate logistic regression to detect the associations between WD and the seven food outlet factors respectively. For the multivariate logistic regression model, based on the published results [12], we adjusted for city (Yuncheng and Suihua), gender, age, educational attainment, family income, and occupation.

Secondly, different age groups might have different demands for the same amenities, leading to different associations of PA to the same neighborhood environment [12,31,55,56,57,58]. To further understand the impact of food outlets on WD among different age groups, participants were categorized into younger adults (aged 18–35) and older adults (aged 36–59). This threshold was the same as that used in previous studies in Chinese contexts [12,59]. We then performed the multivariate logistic regression model for the two age groups.

Finally, using logistic regression, we examined the associations of WD with three levels of food environment diversity, in line with the analysis approach for LUM-diversity [7]. For example, the diversity score can be calculated by averaging rating across the amenity destinations [60], namely, a 7-destination average score in our study. We set three *P* value thresholds of *p* ≤ 0.001, *p* ≤ 0.01, and *p* ≤ 0.05 for the detector sensitivity. Additionally, linear regression was performed as a sensitivity analysis by using the average walking duration on each scale to avoid bias introduced by information loss.

## 3. Results

In the pooled data, 48.31% of participants lived in Yuncheng, 51.69% were male, 52.82% were 18–35 years old, 78.25% had graduated from no higher than junior college (junior college is a post-high school option for people who cannot meet university entry requirements), 42.09% were employed, and 36.72% were “other” (i.e., unemployed or students) (Table 2). Table 3 shows that more than 79% of participants were able to access a market, fruit/vegetable street vending, snack/breakfast street vending, and a grocery store within 10 minutes’ walk. Supermarkets, restaurants, and cafés/tea houses were relatively sporadic in the neighborhood; fewer than 73% of participants could access them within 10 minutes’ walk. Of the seven food amenities, only café/tea house had a relatively high missing value, with 24% of participants not knowing where the amenity was.

### 3.1. Univariate Logistic Regression

The univariate logistic regression produced associations between the seven food environment factors and WD (Table 3), with the association of café/tea house being relatively weak. Market, fruit/vegetable street vending, snack/breakfast street vending, grocery store, supermarket, and restaurant were strongly (*p* ≤ 0.001) related to a longer WD for people who could access these food outlets in 1–5 min compared with those who took more than 10 min. The odds ratio (OR) and 95% confidence interval (CI) were 4.69 (2.37–9.28), 3.28 (1.70–6.32), 7.12 (3.21–15.75), 6.07 (2.48–14.88), 4.09 (2.31–7.24), and 4.96 (2.80–8.78), respectively. Only market and restaurant were strongly (*p* ≤ 0.001) related to a longer WD for people who could access these food outlets in 6–10 min compared with those who took more than 10 min. The OR and 95%CI were 3.56 (1.68–7.53) and 4.46 (2.35–8.48), respectively. In contrast, for café/tea house, only people who lived 6–10 minutes’ walk away reported a longer WD, compared with those who lived more than 10 minutes’ walk away (OR (95%CI): 2.05 (1.08–3.90), *p* ≤ 0.05).

### 3.2. Multivariate Logistic Regression

After adjusting for six potential confounding factors, a longer WD for people who could access the food outlets except for café/tea house in 1–5 min compared with those who took more than 10 min still exist with a decreased trend (Table 3). The OR and 95%CI were 3.42 (1.65–7.08), 2.90 (1.43–5.87), 5.25 (2.25–12.22), 4.67 (1.80–12.08), 2.30 (1.17–4.51), and 3.52 (1.87–6.64) for the market, fruit/vegetable street vending, snack/breakfast street vending, grocery store, supermarket, and restaurant, respectively. People can access market, fruit/vegetable street vending, snack/breakfast street vending, and restaurant within 6–10 min remain to have a longer WD compared to those can access these food outlets more than 10 min. However, for the grocery store, supermarket and café/tea house, the association disappeared after the adjustment for a distance equal to 6–10 minutes’ walk (compared with more than 10 minutes’ walk). The results of the sensitivity analyses were consistent with the results of the univariate and multivariate logistic regressions (Table S1).

### 3.3. Age-Stratified Logistic Regression

The results of this subgroup analysis are shown in Table 4. Café/tea house was not associated with WD in any age group. For people aged 18–35, living 1–5 and 6–10 min away from market and restaurant had a longer WD compared with living more than 10 min away: OR (95%CI): 3.22 (1.08–9.59)/3.44 (1.05–11.32) and 4.79 (1.88–12.16)/4.37 (1.57–12.13). Living 1–5 min away from snack/breakfast street vending and grocery store had also influenced their WD. For people aged 36–59, WD was potentially influenced by most of the food outlet locations, apart from fruit/vegetable street vending, snack/breakfast street vending, and grocery store for 6–10 min away.

### 3.4. Food Environment Diversity

We generated the diversity of residential food outlets by calculating the mean walking time to the seven food outlets (Table 5). A total of 119 participants have a tertile-3 (4.57–5.00), 135 participants have a tertile-2 (3.71–4.43), and 100 participants have a tertile-1 (1.71–3.57). The higher the tertile, the greater the diversity of residential food outlets. Compared with tertile-1, tertile-3, and tertile-2 associated with a longer WD, the ORs (95%CI) were 3.79 (2.16–6.63) and 4.12 (2.27–7.47), respectively, in the univariate model and 3.12 (1.61–6.04) and 2.91 (1.59–5.33), respectively, in the multivariate model.

## 4. Discussion

Walking is one of the most common physical activities across all groups, regardless of social class, gender, or age [13,24]. Due to the fact that most food amenities are close to home (i.e., within 10 minutes’ walk) in low tier Chinese cities, other active travelling modes (e.g., cycling) and the use of public transportation are not common in this study. In the article, we disassembled LUM and only examined the associations between WD and seven types of food-related amenities, taking into account two age groups and three levels of food environment diversity. To our knowledge, and based on a review about environmental correlates of physical activity in China [8], our study constitutes the first quantitative investigation of the walking benefits of food outlets in low tier Chinese cities.

### 4.1. Associations between Food Environments and Physical Activity

Our findings indicate that, apart from café/tea house, the other six food amenities within a short walking distance from home might be meaningful predictors of PA, and that these associations are generally upheld after adjusting for a series of covariates. This suggests that the importance of the availability of neighborhood food outlets as an opportunity to increase PA in small Chinese cities is in line with that revealed by previous studies [28,47]. A potential explanation is that walking distance to the grocery store, supermarket and restaurant is associated with food purchasing, meaning that a shorter walking distance has a higher purchasing motivation [44]. This motivation potentially produces higher-frequency of transport-related walking within a neighborhood.

Notably, the weak association of café/tea house suggests a walking destination for low-frequency recreation with higher consumption. Through field observation in both cities, café houses engaged with younger adults and school children playing mobile phone games or doing homework together, and tea houses attracted groups of people playing mah-jong. Foods had a higher price as optional snacks in café/tea houses, compared with those in other food outlets. Café/tea house naturally belongs to recreational amenities (e.g., karaoke bar) in small Chinese cities that generate higher profits and tend to be located together to facilitate competitiveness, while at the same time not being relevant to regular, basic everyday demands, leading to the non-association of PA [12]. However, an unexpected association of café/tea house became stronger if the duration was 6–10 minutes’ walk from home, compared with a duration of 5 minutes’ walk. This indicates that the associations between walking distance to food outlets and PA do not always adhere to the principle that shorter is better, leading to a critical understanding of the popular attenuation algorithm on walkability [5]. People might occasionally enjoy walking to a café/tea house within walking distance but if it is too close to home other high-demand amenities and necessary activities might be replaced by this more superfluous and recreational amenity. Future studies should examine the nuanced associations of WD with individual food outlets in relation to different demands and motivations.

In general, participants who walked for more than 60 min per day reported less frequently the option “I do not know where the amenity is” (i.e., the “missing” item in Table 3). This suggests that higher WD levels can make people more aware of their neighborhood amenities and, in turn, this awareness might increase their walking motivation and frequency. In addition, more than half of the participants reported a walking distance from home of within 10 min to fruit/vegetable and snack/breakfast street vendors, and these two mobile amenity providers were positively associated with WD. This contributes empirical evidence to the conceptual discussion about the emergence of street vending as a mobile amenity in many residential neighborhoods in China, increasing the LUM and potentially having an impact on walking behavior [14,16,51]. This finding suggests that although street vendors might sell unhealthy food and be loosely controlled by local authorities, they can closely engage with common everyday lives and increase PA in small Chinese cities.

### 4.2. Socioeconomic Factors

The impact of the built environment on walking behavior might be varied by socioeconomic factors [4]. After adjustment, most associations in our study still existed, particularly for market, fruit/vegetable street vending, snack/breakfast street vending, and restaurant. This means that these four food outlets are important destinations within residential neighborhoods, and that they are likely to increase PA for all. In addition, the weak association of café/tea house disappeared, and the associations of supermarket and grocery store became weaker. This reveals that these three outlet types are more variously impacted by different socioeconomic groups. One speculation is that migrants and older people in China prefer to purchase from informal street vendors or markets due to the lower prices, compared with purchasing in supermarkets and grocery stores [12,59]. Our field observation revealed that grocery stores often occurred inside residential buildings at an extremely short distance from home, but the price of fruit and vegetables in these amenities was much higher than when bought from street vendors or in markets, partly due to higher rental costs. Thus, a trade-off is that walking more can expend less money on a similar produce. Future research that examines the association of consumption levels of different food outlets with PA is necessary.

### 4.3. Age Groups and Food Environment Diversity

Other studies have indicated that older people are likely to be affected by the neighborhood environment due to the fact that their bodies are ageing and their time considerably more flexible [56,57]. For this reason, the associations of WD for older adults in our study were stronger and more diverse than those for younger adults. A recent study notes that the strongest association with adolescents’ PA is grocery store [28]. We observed that adults tend to buy cooked foods or raw ingredients to cook for their family, while adolescents generally depend on their parents for meals and prefer to buy snacks from grocery stores. Our results indicated a trend of young adults’ WD being more associated with “cooked foods” (e.g., snack/breakfast street vending and restaurant), while older adults’ WD was more associated with “raw ingredient foods (e.g., fruit/vegetable street vending, market, and supermarket). This reveals that higher consumption outlets with cooked/ready-to-eat foods are likely to increase younger adults’ PA, while basic types of food outlet with lower consumption levels potentially enhance older adults’ PA, in line with the findings from another recent study [12]. In future, research exploring the associations of PA with consumption levels of food outlets should also include age-related differences, particularly in small Chinese cities.

Mixed-use development is a key environmental factor in increasing PA [6,24]; therefore, we hypothesized that combining the seven food amenities together could impact on people’s WD. Following the same analysis [7], our results clearly showed that a higher level of diversity of food outlets in a neighborhood was strongly associated with a longer WD. This demonstrates that if residents in small Chinese cities lived in neighborhoods containing a variety of food outlets, they would be more physically active in their daily lives. Furthermore, apart from café/tea house, the occurrence of the other six food outlets within walking distance from home was high, suggesting that residents subjectively perceived a high availability of food outlets in their current neighborhood. Thus, providing local food outlets is not a critical issue in small Chinese cities, compared with developed countries [28,34]. Additional research is needed to examine the impact of different combinations of individual food outlet on PA, as there might be an “optimal” combination that is more likely to increase walking duration in Chinese neighborhoods.

### 4.4. Local Cultures and Street Vending

Food environments are closely related to local dietary habits in small Chinese cities, which has been shown to be different from western cities. As food outlets are one of the generators for improving physical activity, the nuanced meanings of different food outlets need to be further examined in local contexts, taking into account everyday habits. For instance, the proportion of vegetables consumed in China is higher than in the US, leading to markets and vegetable street vending being important in Chinese everyday lives (Sun et al., 2016; Volpe and Okrent, 2012). As vegetables can be stored for only short periods, Chinese people tend to purchase them frequently and within walking distance, rather than going to a supermarket far from home. Furthermore, informal markets and street vending are often provided by local famers selling products that are both fresh and cheap.

We notice that the different association levels relate to a hierarchy of everyday demands in local contexts. In small Chinese cities, due to rapid development and a lack of strict governance (Liu et al., 2018), food outlets are often closely related to local residents’ real-time demands. For example, our study shows that markets, street vending and restaurants play a more important role than supermarkets and cafés/tea houses (i.e., different associations in Table 3). This reveals that the availability of food outlets in China, in a period of flux and change, are a representation of societal habits and an expression of everyday life. Further studies of the impact of food outlets on physical activity should interpret their results against the wider physical, social and cultural contexts. In other words, the built environment is not just architecture or physical objects; it needs to be understood through the practices of daily living.

During the survey, some participants mentioned that the mobility of street vendors had the potential to temporarily transform the place into an uncertain space in flux. Aside from the low prices, people said that they frequently visited these street spaces in order to see something new or to chat with vendors. For example, one participant visited the street vending area to see whether there were any new vendors selling new kinds of fruit (e.g., grapes, which were in season during the survey period). Another potential mechanism between street vending and increased WD might be in relation to the enhancement of social connectedness on a daily basis, namely, seeing fresh actors and chatting with vendors might contribute to a change in mood and potentially help restore people’s mental well-being [29]. Future research should examine transport-related PA in tandem with an examination of the health benefits of street vending.

### 4.5. Strengths and Limitations

Some caution is required in interpreting the results of this study. The cross-sectional approach cannot confirm causality between residential food outlets and WD. Due to different health conditions and disabilities, perceived walking duration might differ from the actual walking time measured by objective pedometers. For example, compared with younger people, older people are likely to perceive a longer walk for the same distance [55,57]. However, this potential deviation has been reduced by our exclusion of people over the age of 59. For further studies, other residual or unmeasured confounding related to environmental factors such as safety and street quality should be included. Given the inclusion of only a small number of food outlet typologies, an exploration of the potential health benefits of other food outlets such as bars and food delivery providers is warranted in future studies. In addition to studying only static food amenities such as cafés and restaurants, the physical health benefits of the mobile food outlets such as street vending need to be separately studied. Other health factors such as health status or disability also need to be considered.

Nevertheless, this study has its advantages, in that it expands existing knowledge of neighborhood walkability by providing empirical evidence of the fine-grained relationship between food outlets and WD, in small Chinese cities, where a majority of the Chinese population live. A few studies have shown that type of commercial food destination is differently correlated with physical activity/walking across different age groups [28]. Our study not only provides detailed evidence of how the proximity of neighborhood food destinations increases PA; it also explores nuanced associations for younger and older adults. Although such age-related differences were not particularly remarkable, a latent trend related to cooked foods and raw ingredients was uncovered.

Additionally, street vending practices should be further facilitated by Chinese city authorities, as they have been shown in this study to be a potential built environment construct that enhances walking duration. How different categories of time-related street vending are associated with nearby residents’ PA or other health outcomes could be a key area for future research. Scholars also need to explore the hierarchy of different food outlet typologies concerning local culture and society in Chinese contexts. Overall, this novel study reveals a series of potential research directions that deserve more detailed examination in the future.

## 5. Conclusions

Returning to the research questions in this study, our findings showed that the individual and the diversity of food amenities within a shorter walking distance from home were associated with a higher self-rated daily walking duration in low tier Chinese cities. There were no major differences between the two age groups (i.e., 18–35 and 36–59). However, there needs to be a differentiation between necessary and recreational food amenities in local neighborhoods, because the two typologies are likely to play different roles in increasing PA. Some food outlets with a recreational purpose and high consumption levels need to be carefully considered since they might engage mainly younger people, which might decrease the common residents’ PA. In short, although some recent studies note that using the LUM to promote PA is ineffective in high-density Chinese cities, residential food outlets as one of the land-use categories are positively associated with PA in small Chinese cities, including the mobile and informal street vendors for fruit/vegetable and snack/breakfast.

## Figures and Tables

**Table 1:** Land use classification in China (GB/T21010–2017), published by Ministry of Land and Resources in 2017 [32].

Current Land Use Classification	
1	Arable land
2	Garden
3	Woodland
4	Natural grassland
5	Commercial land
6	Industrial and mining warehouse
7	Residential land
8	Land for public administration and public services
9	Special land
10	Transportation land
11	Land for water and water conservancy facilities
12	Other

**Table 2:** Descriptive analysis of sociodemographic characteristics.

Sociodemographic Characteristics	All	
	*N*	%
*City*		
Yuncheng	171	48.31
Suihua	183	51.69
*Gender*		
Male	183	51.69
Female	171	48.31
*Age groups*		
18–35	187	52.82
36–59	167	47.18
*Educational attainment*		
Junior College or below	277	78.25
Bachelor or higher	77	21.75
*Occupation*		
Employed	149	42.09
Self-employed	75	21.19
Others	130	36.72
*Family income (Chinese Yuan)*		
3000 or below	80	22.60
3001–5000	131	37.01
5001+	143	40.40

**Table 3:** Logistic regression analysis for the associations between residential food environment variables and perceived daily walking duration.

Walking Distance	Perceived Daily Walking Duration ^a^					
	*N*/%		Univariate		Multivariate ^b^	
	>60	≤60	OR (95% CI)	*p*-Value	OR (95% CI)	*p*-Value
(Fruit/vegetable) market						
1–5 min	91/26%	97/27%	4.69 (2.37–9.28)	0.000 ***	3.42 (1.65–7.08)	0.001 ***
6–10 min	37/10%	52/15%	3.56 (1.68–7.53)	0.001 ***	3.33 (1.50–7.36)	0.003 **
>10 min	12/3%	60/17%	1		1	
Missing	1/0%	4/1%				
Fruit/vegetable street vending						
1–5 min	91/26%	101/29%	3.28 (1.70–6.32)	0.000 ***	2.90 (1.43–5.87)	0.003 **
6–10 min	36/10%	60/17%	2.19 (1.06–4.50)	0.034 *	2.37 (1.09–5.14)	0.029 *
>10 min	14/4%	51/14%	1		1	
Missing	0/0%	1/0%				
Snack/breakfast street vending						
1–5 min	104/29%	95/27%	7.12 (3.21–15.75)	0.000 ***	5.25 (2.25–12.22)	0.000 ***
6–10 min	27/8%	62/18%	2.83 (1.18–6.76)	0.019 *	3.41 (1.36–8.55)	0.009 **
>10 min	8/2%	52/15%	1		1	
Missing	2/1%	4/1%				
(Convenience/small) grocery store						
1–5 min	113/32%	121/34%	6.07 (2.48–14.88)	0.000 ***	4.67 (1.80–12.08)	0.001 ***
6–10 min	22/6%	53/15%	2.70 (1.00–7.28)	0.050 *	2.12 (0.74–6.07)	0.159
>10 min	6/2%	39/11%	1		1	
Missing	0/0%	0/0%				
Supermarket						
1–5 min	90/25%	87/25%	4.09 (2.31–7.24)	0.000 ***	2.30 (1.17–4.51)	0.016 *
6–10 min	31/9%	46/13%	2.66 (1.36–5.20)	0.004 **	2.04 (0.99–4.22)	0.054
>10 min	20/6%	79/22%	1		1	
Missing	0/0%	1/0%				
Restaurant						
1–5 min	77/22%	71/20%	4.96 (2.80–8.78)	0.000 ***	3.52 (1.87–6.64)	0.000 ***
6–10 min	40/11%	41/12%	4.46 (2.35–8.48)	0.000 ***	3.87 (1.95–7.66)	0.000 ***
>10 min	21/6%	96/27%	1		1	
Missing	3/1%	5/1%				
Café/tea house						
1–5 min	18/5%	19/5%	2.03 (0.99–4.14)	0.053	1.45 (0.65–3.21)	0.363
6–10 min	24/7%	25/7%	2.05 (1.08–3.90)	0.028 *	1.44 (0.70–2.96)	0.318
>10 min	58/16%	124/35%	1		1	
Missing	41/12%	45/13%				

OR: odds ratio; CI: confidence interval. ^a^ >60 min versus ≤60 min (referent). ^b^ Analyses adjusted for city (Yuncheng, Suihua), gender (male, female), age groups (18–35, 36–59), education attainment (junior college or below, bachelor or higher), household income (3000 or below, 3001–5000, 5001+), occupation (employed, self-employed, others). * *p* ≤ 0.05, ** *p* ≤ 0.01, *** *p* ≤ 0.001.

**Table 4:** Logistic regression analysis for the associations between residential food environment variables and perceived daily walking duration by age groups.

Walking Distance	Perceived Daily Walking Duration ^a^							
	Aged 18–35 (*n* = 187)				Aged 36–59 (*n* = 167)			
	*N*/%		Multivariate ^b^		*N*/%		Multivariate ^b^	
	>60	≤60	OR (95% CI)	*p*-Value	>60	≤60	OR (95% CI)	*p*-Value
(Fruit/vegetable) market								
1–5 min	38/20%	55/29%	3.22 (1.08–9.59)	0.036 *	53/32%	42/25%	3.78 (1.39–10.33)	0.009 **
6–10 min	18/10%	32/17%	3.44 (1.05–11.32)	0.042 *	19/11%	20/12%	3.12 (1.04–9.30)	0.041 *
>10 min	5/3%	35/19%	1		7/4%	25/15%	1	
Missing	1/1%	3/2%			0/0%	1/1%		
Fruit/vegetable street vending								
1–5 min	40/21%	62/33%	2.71 (0.90–8.21)	0.078	51/31%	39/23%	3.22 (1.25–8.27)	0.015 *
6–10 min	17/9%	33/18%	2.79 (0.83–9.35)	0.097	19/11%	27/16%	1.90 (0.66–5.47)	0.232
>10 min	5/3%	29/16%	1		9/5%	22/13%	1	
Missing	0/0%	1/1%			0/0%	0/0%		
Snack/breakfast street vending								
1–5 min	46/25%	52/28%	7.13 (1.51–33.56)	0.013 *	58/35%	43/26%	5.24 (1.77–15.51)	0.003 **
6–10 min	14/7%	44/24%	4.63 (0.92–23.31)	0.063	13/8%	18/11%	2.88 (0.86–9.57)	0.085
>10 min	2/1%	26/14%	1		6/4%	26/16%	1	
Missing	0/0%	3/2%			2/1%	1/1%		
(Convenience/small) grocery store								
1–5 min	49/26%	66/35%	6.45 (1.34–30.93)	0.020 *	64/38%	55/33%	3.71 (1.08–12.81)	0.038 *
6–10 min	11/6%	36/19%	2.35 (0.43–12.80)	0.322	11/7%	17/10%	1.90 (0.47–7.73)	0.368
>10 min	2/1%	23/12%	1		4/2%	16/10%	1	
Missing	0/0%	0/0%			0/0%	0/0%		
Supermarket								
1–5 min	37/20%	45/24%	1.98 (0.75–5.22)	0.169	53/32%	42/25%	2.90 (1.09–7.74)	0.034 *
6–10 min	16/9%	33/18%	1.48 (0.53–4.10)	0.455	15/9%	13/8%	3.11 (1.06–9.08)	0.038 *
>10 min	9/5%	46/25%	1		11/7%	33/20%	1	
Missing	0/0%	1/1%			0/0%	0/0%		
Restaurant								
1–5 min	35/19%	37/20%	4.79 (1.88–12.16)	0.001 ***	42/25%	34/20%	3.08 (1.19–7.96)	0.020 *
6–10 min	18/10%	27/14%	4.37 (1.57–12.13)	0.005 **	22/13%	14/8%	3.88 (1.45–10.37)	0.007 **
>10 min	8/4%	58/31%	1		13/8%	38/23%	1	
Missing	1/1%	3/2%			2/1%	2/1%		
Café/tea house								
1–5 min	10/5%	11/6%	1.93 (0.62–5.97)	0.253	8/5%	8/5%	0.88 (0.27–2.92)	0.834
6–10 min	15/8%	13/7%	2.31 (0.83–6.41)	0.107	9/5%	12/7%	0.91 (0.32–2.64)	0.867
>10 min	26/14%	75/40%	1		32/19%	49/29%	1	
Missing	11/6%	26/14%			30/18%	19/11%		

OR: odds ratio; CI: confidence interval. ^a^ >60 min versus ≤60 min (referent). ^b^ Analyses adjusted for city (Yuncheng, Suihua), gender (male, female), age groups (18–35, 36–59), education attainment (junior college or below, bachelor or higher), household income (3000 or below, 3001–5000, 5001+), occupation (employed, self-employed, others). * *p* ≤ 0.05, ** *p* ≤ 0.01, *** *p* ≤ 0.001.

**Table 5:** Logistic regression analysis for the association between the diversity of residential food environment variables and perceived daily walking duration.

Diversity Level	Perceived Daily Walking Duration					
	*N*		Unadjusted		Adjusted ^a^	
	>60	≤60	OR (95% CI)	*p*-Value	OR (95% CI)	*p*-Value
Diversity of residential food constructs ^b^						
Tertile 3 (4.57–5.00)	24	95	3.79 (2.16–6.63)	0.000	3.12 (1.61–6.04)	0.001
Tertile 2 (3.71–4.43)	66	69	4.12 (2.27–7.47)	0.000	2.91 (1.59–5.33)	0.001
Tertile 1 (1.71–3.57)	51	49	1		1	

OR: odds ratio; CI: confidence interval. ^a^ Analyses adjusted for city (Yuncheng, Suihua), gender (male, female), age groups (18–35, 36–59), education attainment (junior college or below, bachelor or higher), household income (3000 or below, 3001–5000, 5001+), occupation (employed, self-employed, others). ^b^ Diversity of residential food constructs = average walking time to the seven food outlets (5 = 1–5 min; 4 = 6–10 min; 3 = 11–20 min; 2 = 21–30 min; 1 = 31+ minutes and don’t know). A “don’t know” response is also coded as “1” because the walking distance to an unaware amenity is likely more than 31 min.

## Data Availability

The data that support the findings of this study are available from EThOS (DOI:10.7488/era/255), but restrictions apply to the availability of these data until 02.07.2021, and so are not publicly available yet.

## Supplementary Materials

The following are available online at https://www.mdpi.com/1660-4601/18/2/839/s1, Table S1: Linear regression analysis for the associations between residential food environment variables and perceived daily walking duration.
