# Supplementary material for: Time Distances to Residential Food Amenities and Daily Walking Duration: A Cross-Sectional Study in Two Low Tier Chinese Cities
Source: Int J Environ Res Public Health. 2021 Jan 19;18(2):839. doi: 10.3390/ijerph18020839 (PMC7844623; doi:10.3390/ijerph18020839)
Supplement: Supplementary file 1 [file ijerph-18-00839-s001.pdf]

Table S1. Linear regression analysis for the associations between residential food environment variables and perceived daily walking duration.

Walking Distance	Perceived Daily Walking Duration [a]				
	Unadjusted			Adjusted [b]	
	N	Coefficient (S.E.)	*p*-Value	Coefficient (S.E.)	*p*-Value
(Fruit/vegetable) market					
1–5 minutes	188	33.67 (7.17)	0.000 ***	21.68 (7.26)	0.003 **
6–10 minutes	89	23.95 (8.20)	0.004 **	18.43 (8.05)	0.023 *
>10 minutes	72	1		1	
Missing	5				
Fruit/vegetable street vending					
1–5 minutes	192	28.33 (7.56)	0.000 ***	21.33 (7.28)	0.004 **
6–10 minutes	96	16.45 (8.44)	0.052	15.74 (8.05)	0.051
>10 minutes	65	1		1	
Missing	1				
Snack/breakfast street vending					
1–5 minutes	199	40.26 (7.41)	0.000 ***	29.44 (7.65)	0.000 ***
6–10 minutes	89	7.92 (8.42)	0.348	10.44 (8.28)	0.208
>10 minutes	60	1		1	
Missing	6				
(Convenience/small) grocery store					
1–5 minutes	234	37.38 (8.39)	0.000 ***	25.41 (8.35)	0.003 **
6–10 minutes	75	15.03 (9.72)	0.123	8.29 (9.47)	0.382
>10 minutes	45	1		1	
Missing	0				
Supermarket					
1–5 minutes	177	32.02 (6.46)	0.000 ***	18.69 (7.39)	0.012 *
6–10 minutes	77	22.01 (7.85)	0.005 **	16.13 (7.89)	0.042 *
>10 minutes	99	1		1	
Missing	1				
Restaurant					
1–5 minutes	148	35.01 (6.25)	0.000 ***	23.94 (6.68)	0.000 ***
6–10 minutes	81	37.82 (7.23)	0.000 ***	29.28 (7.22)	0.000 ***
>10 minutes	117	1		1	
Missing	8				
Café/tea house					
1–5 minutes	37	8.90 (9.48)	0.349	0.95 (8.92)	0.915
6–10 minutes	49	6.34 (8.14)	0.437	−1.12 (8.01)	0.888
>10 minutes	182	1		1	
Missing	86				

S.E.: Standard deviation. [a] Recalibrations of the scale with mid-points: 1–10 minutes = 5 minutes; 11–30 minutes = 20 minutes; 31–60 minutes = 45 minutes; 61–120 minutes = 90 minutes; 121 minutes+ = 180 minutes. [b] Analyses adjusted for city (Yuncheng, Suihua), gender (male, female), age groups (18–35, 36–59), education attainment (junior college or below, bachelor or higher), household income (3000 or below, 3001–5000, 5001+), occupation (employed, self-employed, others). * $p \leq 0.05$, ** $p \leq 0.01$, *** $p \leq 0.001$.
